# Value of Different Comorbidity Indices for Predicting Outcome in Patients with Acute Myeloid Leukemia

**DOI:** 10.1371/journal.pone.0164587

**Published:** 2016-10-12

**Authors:** Maxi Wass, Friederike Hitz, Judith Schaffrath, Carsten Müller-Tidow, Lutz P. Müller

**Affiliations:** Department of Hematology and Oncology, University Hospital Halle, Halle/Saale, Germany; European Institute of Oncology, ITALY

## Abstract

Age is a dominant predictor of outcome in acute myeloid leukemia (AML). However, it is not clear to which extent comorbidities contribute to this effect. The objective of this study was to determine the impact of pretreatment comorbidities on survival of AML patients. In a single-center retrospective study 194 adult AML patients were included. The Hematopoietic cell transplantation comorbidity index (HCT-CI), the Adult Comorbidity Evaluation-27 (ACE-27) score and the Cumulative Illness Rating Scale for Geriatrics (CIRS-G) as well as data on demographics, cytogenetics, treatment and outcome were evaluated at the time of initial diagnosis by univariate and multivariate analysis. The study included 102 male and 92 female (median age 60.9 years) of which 173 (89.2%) received intensive chemotherapy. Median overall survival (OS) was 17 months. In univariate analysis, cardiovascular disease (26 vs 12 months, *p* = .005), severe hepatic disease (19 vs 4 months, *p* = .013) and renal impairment (17 vs 7 months, *p* = .016) was associated with inferior OS. For each index, the highest comorbidity burden was associated with reduced OS. However, in multivariate analysis only the ACE-27 score was associated with outcome. Besides ECOG ≥ 2 and poor cytogenetics only the ACE-27 score but not higher age was associated with OS in the group of patients receiving intensive therapy. Adjusted hazard ratios were 3.1, 3.5 and 4.0 for mild, moderate and severe ACE-27-assessed comorbidities, respectively (*p* = .012). Our study confirms that comorbidities significantly impact survival of AML patients and a pretreatment assessment of comorbidities may help to identify patients with poor outcome.

## Introduction

Acute myeloid leukemia (AML) is a heterogeneous hematopoietic disease and depending on risk factors survival and prognosis is still poor [1]. Along with disease-related factors, such as cytogenetics [2] and specific molecular changes [3,4], patient-related factors particularly age and performance status [5,6] are strong predictors for outcome allocating treatment decisions [7]. Recently, a specific AML risk-score has been developed to estimate the chance to achieve complete remission (CR) and calculate the risk of early death (ED) after intensive induction therapy in patients aged older 60 years with previously untreated AML [8]. Body temperature, age, de-novo leukemia versus secondary leukemia, hemoglobin, platelet count, fibrinogen and serum lactate dehydrogenase (LDH) were significantly associated with CR and ED [8]. However, the AML score did not include performance status, comorbidities, or severe organ failure. Older age has been demonstrated as a consistent adverse prognostic factor in AML. However, single as well as accumulated comorbidities that lower tolerance to chemotherapy may contribute to poorer outcome in older patients.

Several reports support the assumption that comorbidities have a significant prognostic influence on the outcome of patients with AML [9–12]. Specifically the Hematopoietic cell transplantation comorbidity index (HCT-CI) can predict non-relapse mortality and overall survival for AML patients post stem cell transplantation (SCT) [13,14]. Assessment of comorbidities by HCT-CI or the Adult Comorbidity Evaluation-27 (ACE-27) score has demonstrated a significant prognostic influence on the survival of patients with AML [9–11] and MDS [15,16]. However, these data were almost exclusively derived in elderly patients. Conclusive data of the impact of concomitant diseases on the outcome of AML in general and of the differential value of various comorbidity indices are sparse. Thus, risk stratification for AML at diagnosis is currently primarily based on cytogenetics, performance status and chronological age without including comorbidity assessment [8,17–19].

We hypothesize that assessment of comorbidities may better reflect biological age and may therefore help to evaluate prognosis at time of initial diagnosis.

The object of this study was to determine the impact of comorbidities on the survival of patients with AML and to compare three validated comorbidity indices in predicting outcome at the time of initial diagnosis.

## Patients and Methods

### Patients and assessment of comorbidities

We conducted a retrospective cohort study of 194 adult patients with newly diagnosed AML who presented at the Department of Internal Medicine IV, University hospital of Halle between 1996 and 2012. Information on present comorbidities at the time of initial diagnosis before treatment initiation was retrieved from the medical files. The following three indices were used to categorize the patients: the HCT-CI, the ACE-27 score and the Cumulative Illness Rating Scale for Geriatrics (CIRS-G). In brief, for the HCT-CI, patients were categorized based on the sum of weighted comorbidities into the following three groups: score of 0 (low), 1–2 (intermediate), and ≥ 3 (high), as previously reported [13]. For the ACE-27 score, each patient was given an overall grade of none, mild, moderate, or severe comorbidity, as previously described [20]. Finally, for the CIRS-G patients were placed into one of the following three groups corresponding to the total sum of weighted comorbidities (TSC): TSC of 0 (low), ≤ 6 (intermediate), and > 6 (high), as previously reported [21].

In addition to demographic data, including age at diagnosis and sex, we collected the following disease-related factors and clinical data: bone marrow blasts, serum LDH, peripheral blood leukocytes, AML subtype classification by the French-American-British (FAB) cooperative group [22], cytogenetic risk groups using the European LeukemiaNet (ELN) recommendations [1] and performance status using the Eastern Cooperative Oncology Group (ECOG) scale. Additionally, we documented the date of initial diagnosis, each patient´s treatment and the date of last contact or death.

All patients had consented to treatment according to local standards. Data were handled pseudonymised during all stages of accrual and analysis. Given this pseudonymised analysis of retrospectively accrued data no reactive consent or Institutional Review Board (IRB) approval was deemed necessary.

### Statistical Analysis

The primary end point of this study was overall survival (OS), calculated in months from initial diagnosis until death of any cause. Observation was censored for patients alive at last follow up. For subgroup analysis early death (ED) was defined as death within 28 days after start of chemotherapy; complete remission (CR) was defined according to standard criteria [1]. Standard descriptive statistics were used to analyze the study population. To demonstrate the prognostic impact of comorbidity on survival, we used Kaplan-Meier methods, and differences in survival between groups were tested using the long-rank test. Univariate analysis for the influence of potential prognostic factors on ED were performed using the x² test. Variables reaching statistical significance (*p* < 0.05) in univariate analysis were included in the multivariate analysis using the backward selection method. Logistic regression models were used for multivariate analysis of prognostic variables for ED and cox proportional hazard models were used for analysis of survival. The significance level was 0.05 in the multivariate analysis. Hazard ratios were calculated with a 95% confidence interval (CI). All statistical analysis were performed using SPSS (version 22 for Windows).

## Results

### Patient characteristics

A total of 194 adult AML patients was included in the study. Median duration of follow-up was 9 months (range, 0–181). Patient characteristics are listed in Table 1. The median age at diagnosis was 60.9 years (range, 18–90); more than the half of patients was older than 60 years. Sex distribution was equal. Karyotype was available for 144 patients (74.2%). One hundred seventy three (89.2%) patients received intensive chemotherapy, 43 (22.2%) underwent SCT and 112 (57.7%) patients died during follow up. Intensive induction chemotherapy consisted of standard anthracycline-based chemotherapy (conventional “7+3” regimen, i.e. anthracycline and cytarabine). About two-thirds (72.8%) intensively treated patients achieved complete remission (CR).

**Table 1:** Patient characteristics.

Variable		Total no. of patients (%)
Age, years	< 60	75 (38.7)
	≥ 60	119 (61.3)
Sex	Male	102 (52.6)
	Female	92 (47.4)
AML type	De novo	123 (63.4)
	Secondary	71 (36.6)
Cytogenetics	Good	19 (13.2)
	Intermediate	87 (60.4)
	Poor	38 (26.4)
Regimen	Intensive CTx	173 (89.2)
	Palliative CTx	21 (10.8)
SCT	No	151 (77.8)
	Yes	43 (22.2)
ECOG	0–1	131 (81.9)
	≥ 2	29 (18.1)
CR	Yes	126 (72.8)
	No	44 (25.4)

CTx, chemotherapy; CR, complete remission; ECOG, Eastern Cooperative Oncology Group; SCT, stem cell transplantation.

### Distribution of comorbidities

**Fig 1:**
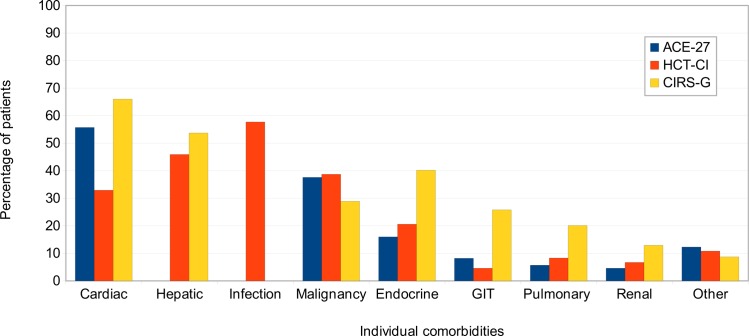
Distribution of individual comorbidities. Individual comorbidities among 194 AML patients as assessed by the three indices, respectively. Hepatic diseases are not assessed by the ACE-27 score, and infections are only assessed by the HCT-CI.

We first analyzed the distribution of individual comorbidities according to the three indices in order to evaluate their sensitivity in assessing concomitant disease burden. In general, the most frequent comorbidities were cardiovascular disease (59.8%), with hypertension being the most common specific ailment (52.1%), followed by infection (57.7%), hepatic disorders (45.9%) and prior malignancy (39.2%) (Fig 1). The HCT-CI and ACE-27 score were comparable in capturing prior malignancy, endocrine, gastrointestinal, respiratory and renal comorbidities, but often with lower frequency than the CIRS-G. Higher percentage of infection and hepatic comorbidity were detected by the HCT-CI and CIRS-G compared with the ACE-27 score, while cardiovascular comorbidity was lowest in the HCT-CI. Almost no differences were seen between the three scores in defining other comorbidities, including neurologic, psychiatric and rheumatologic impairments. Most patients had moderate / intermediate or severe comorbidity, although differences were apparent based on the categorization for each index (Table 2). Notably, the frequency of the severity of comorbidity was more homogeneously distributed using the ACE-27 score compared with the other two indices (Table 2).

**Table 2:** Distribution of comorbidities.

Comorbidity category according to the respective index		Total no. of patients (%)
ACE-27 score	None, 0	39 (20.1)
	Mild, 1	50 (25.8)
	Moderate, 2	74 (38.1)
	Severe, 3	31 (16.0)
HCT-CI	Low, 0	14 (7.2)
	Intermediate, 1–2	61 (31.4)
	High, ≥ 3	119 (61.3)
CIRS-G	Low, 0	12 (6.2)
	Intermediate, 1–6	96 (49.5)
	High, > 6	86 (44.3)

ACE-27, Adult Comorbidity Evaluation-27; HCT-CI, Hematopoetic cell transplantation comorbidity index; CIRS-G, Cumulative Illness Rating Scale for Geriatrics.

### Impact of comorbidities and general risk factors on survival in the entire patient cohort

**Fig 2:**
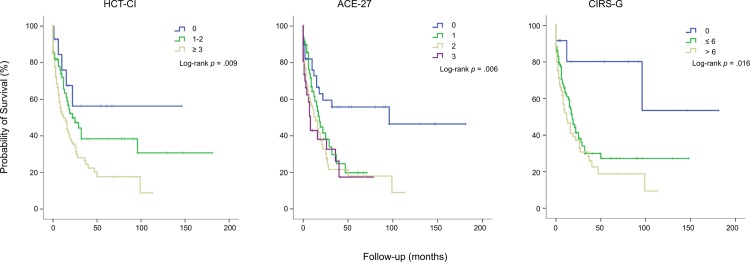
Survival curves according to the 3 comorbidity indices HCT-CI, ACE-27 and CIRS-G. Risk groups based on comorbidity were stratified by HCT-CI, ACE-27 score and CIRS-G in all 194 patients and differences in survival between groups were tested using the long-rank test.

For each index, the highest comorbidity burden was associated with worst overall survival (OS; Fig 2). For the HCT-CI, median OS was not reached for patients in the low risk group (0 points) and 22 and 12 months for patients in the intermediate- (1–2 points) and high- (≥ 3 points) risk group, respectively (*p =* .009). Median OS according to the ACE-27 score was 96, 18, 14 and 8 months for patients with none, mild, moderate and severe comorbidities, respectively (*p* = .006). According to the CIRS-G median OS was not reached, 17 and 12 months for patients in the low- (0 points), intermediate- (1–6 points) and high- (> 6 points) risk group, respectively (*p* = .016).

Cardiovascular disease was associated with inferior OS when assessed according to the ACE-27 score (26 vs 12 months, *p =* .005), HCT-CI (18 vs 12 months, *p =* .003) and CIRS-G (25 vs 12 months, *p =* .001). Also renal impairment was associated with inferior survival according to the ACE-27 score (17 vs 12 months, *p =* .016), HCT-CI (18 vs 12 months, *p* = .003) and CIRS-G (18 vs 12 months, *p* = .050). According to the HCT-CI, but not CIRS-G, severe hepatic disorder was associated with shorter survival (19 vs 4 months, *p* = .013). The ACE-27 score does not assess hepatic disease.

We also determined median OS according to different patient-, treatment- and disease-related factors (S1 Table): older age, poor cytogenetics and ECOG ≥ 2 were significantly associated with reduced OS, whereas intensive chemotherapy and subsequent performance of SCT significantly correlated with longer OS. Additionally, higher peripheral blood leukocytes and serum LDH at initial diagnosis was correlated with shorter OS, whereas sex, bone marrow blasts or AML type (de novo vs secondary) were not associated with OS in this univariate analysis.

Multivariate cox proportional regression models were conducted to assess whether the presence of comorbidities according to each index was independently associated with OS (Table 3). These models were adjusted for all those covariates which showed significance in univariate analysis. Among the three comorbidity indices only categorization according to the ACE-27 score was significantly associated with OS in multivariate analysis. Compared to the group of patients with no comorbidity according to the ACE-27 score, adjusted hazard ratios for OS were 2.1, 2.3 and 4.3 for patients with mild, moderate and severe comorbidities, respectively. In addition, ECOG ≥ 2 at presentation and poor cytogenetics were significantly associated with OS. Interestingly, higher age (i. e. ≥ 60 years) had no significant impact on OS in multivariate analysis.

**Table 3:** Cox proportional hazard regression model with factors predicting overall survival for the entire cohort.

	univariate			multivariate		
Variable	HR	95% CI	*p*	HR*	95% CI	*p*
ACE-27 score						
None, 0	Ref			Ref		
Mild, 1	1.91	1.03–3.55	.041	2.05	0.82–5.13	NS
Moderate, 2	2.59	1.46–4.60	.001	2.30	1.00–5.32	.050
Severe, 3	2.74	1.41–5.34	.003	4.30	1.63–11.32	.003
Cytogenetics						
Good	Ref			Ref		
Intermediate	1.53	0.69–3.40	NS	1.72	0.64–4.59	NS
Poor	3.01	1.30–6.97	.010	4.40	1.62–11.93	.004
ECOG						
0–1	Ref			Ref		
≥ 2	3.18	1.96–5.16	.000	3.12	1.47–6.61	.003
Age, years						
< 60	Ref			Ref		
≥ 60	1.82	1.22–2.71	.003	1.53	0.84–2.78	NS
LDH, U/l						
≤ 700	Ref			Ref		
> 700	2.17	1.48–3.19	.000	1.28	0.70–2.34	NS
WBC, /L						
≤ 30x10^9^	Ref			Ref		
> 30x10^9^	1.57	1.07–2.28	.020	1.51	0.90–2.64	NS

HR, hazard ratio; CI, confidence interval; ACE-27, Adult Comorbidity Evaluation-27; ECOG, Eastern Cooperative Oncology Group; WBC, white blood cell; LDH, lactate dehydrogenase; NS, not significant; Ref, reference.

*HR was adjusted to age, ACE-27, ECOG, cytogenetics, WBC and LDH.

Survival analysis (Fig 2) suggested that a reduced OS was associated with the presence of at least ACE-27-classified comorbidity rather than with ACE-27 subgroups. Therefore the multivariate model was applied for the entire cohort using a consolidated dichotomous ACE-27 grouping “no” (ACE-27 score 0) vs “at least one comorbidity” (ACE-27 score >0). However, results were similar with presence of ACE-27 comorbidity, poor cytogenetics and ECOG ≥ 2 showing a significant impact on OS (S2 Table).

In addition CR rate was significantly associated with longer OS (27 vs 2 months, *p* = .000). However, the aim of the study was to identify factors of comorbidity which when assessed at initial diagnosis prior to therapy may aid decision on treatment strategy. Therefore CR rate was deliberately not included in primary uni- and multivariate analysis. When CR rate was included it expectedly showed a significant impact on OS in uni- and multivariate analysis (univariate: HR 5.19; CI 3.46–7.81; *p* = .000; multivariate: HR 4.46; CI 2.30–8.65; *p* = .000).

### Impact of comorbidities on early death and overall survival in patients with intensive induction chemotherapy

**Fig 3:**
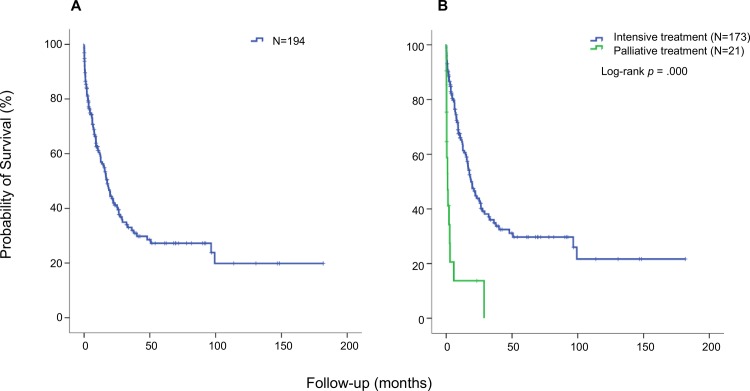
Overall survival for the entire patient cohort and for different treatment strategies. Kaplan-Meier analysis was performed and the effect of treatment on OS was tested using the long-rank test. **(A)** OS in the entire patient cohort. The median OS was 17 months. **(B)** OS in the patient groups according to treatment strategy. The median OS of the 173 patients treated intensively was 18 months (blue line) while the 21 patients who received palliative treatment, had a median OS of one month (green line).

When treatment-related factors were included in our univariate analysis (S1 Table; Fig 3) choice of therapy strategy (palliative vs intensive chemotherapy) as well as subsequent SCT had a significant impact on survival. Choice of treatment likely reflects the subjective assessment of comorbidities by the treating physician. Analysis of the impact of comorbidities in the entire patient group will therefore be biased. Thus, we analyzed the impact of comorbidities on early death (ED) and OS only for patients having received intensive induction chemotherapy (Table 4).

**Table 4:** Factors associated with early death (ED) and overall survival (OS) in patients with intensive induction chemotherapy.

		ED Rate (N = 17)					OS (N = 173)		
		univariate		multivariate			multivariate		
Variable		N (%)	*p*^*a*^	OR*	95% CI	*p*^*b*^	HR*	95% CI	*p*^*c*^
Age, years	< 60	5.6					Ref		
	≥ 60	12.9	.117	—	—	—	1.05	0.58–1.90	NS
ACE-27 score	None	4.9		Ref			Ref		
	Mild						3.11	1.28–7.52	.012
	Moderate	14.4	.036	3.19	0.78–13.05	NS	3.50	1.46–8.37	.005
	Severe						4.02	1.42–11.39	.009
Cytogenetics	Good	—					Ref		
	Intermediate	6.3					2.13	0.82–5.53	NS
	Poor	9.4	NS	—	—	—	4.78	1.74–13.12	.002
ECOG	0–1	6.3		Ref			Ref		
	≥ 2	25.0	.007	4.63	1.21–17.75	.025	1.78	0.73–4.31	NS
SCT	Yes						Ref		
	No	—		—	—	—	2.42	1.31–4.47	.005
LDH, U/l	≤ 700	4.8		Ref			Ref		
	> 700	18.6	.004	3.19	0.97–13.40	NS	1.06	0.52–2.13	NS
WBC, /L	≤ 30x10^9^	7.3							
	> 30x10^9^	14.5	NS	—	—	—	—	—	—
AML type	De novo	9.8							
	Secondary	10.0	NS	—	—	—	—	—	—

ACE-27, Adult Comorbidity Evaluation-27; ECOG, Eastern Cooperative Oncology Group; SCT, stem cell transplantation; WBC, white blood cell; LDH, lactate dehydrogenase; OR, odds ratio; HR, hazard ratio; Ref, reference; NS, not significant.—indicates not applicable.

^*a*^ *p-*value from x² test

^b^ *p*-value from multivariate logistic regression

^c^ *p*-value from multivariate cox regression.

*OR and *HR were adjusted for risk factors reaching statistical significance in univariate analysis (*p*<0.05).

Seventeen of 173 patients treated with intensive chemotherapy succumbed to ED. Due to the low number of patients with ED, ACE-27 risk groups were combined for the following analysis. There was a significant difference in ED rate between patients with none or mild versus moderate and severe comorbidities (*p* = .036; Table 4). Additional risk factors for ED were: ECOG ≥ 2 (*p* = .007) and elevated LDH (*p* = .004) at initial diagnosis. In multivariate logistic regression only ECOG ≥ 2 (*p* = .025) was an independent risk factor for ED.

In contrast, for OS multivariate analysis revealed that, as for the entire patient group, poor cytogenetics and presence of severe comorbidities according to ACE-27 score as well as subsequent performance of SCT have a significant and independent impact on survival in intensively treated patients. Interestingly contrary to ED, ECOG and age had no significant impact on OS (Table 4).

As for the entire cohort the multivariate model using a consolidated dichotomous ACE-27 grouping (ACE-27 score 0 vs >0) was applied for the intensively treated cohort. Results were similar with presence of ACE-27 comorbidity, poor cytogenetics and performance of SCT showing a significant impact on OS (S2 Table).

Again, CR rate was deliberately not included in the primary analysis in that CR rate is not available as a prognostic factor at initial diagnosis. When CR rate was included in multivariate analysis it had a significant impact on OS (HR 4.40; CI 2.13–9.05; *p* = .000).

### Relation of age and ACE-27 distribution

**Fig 4:**
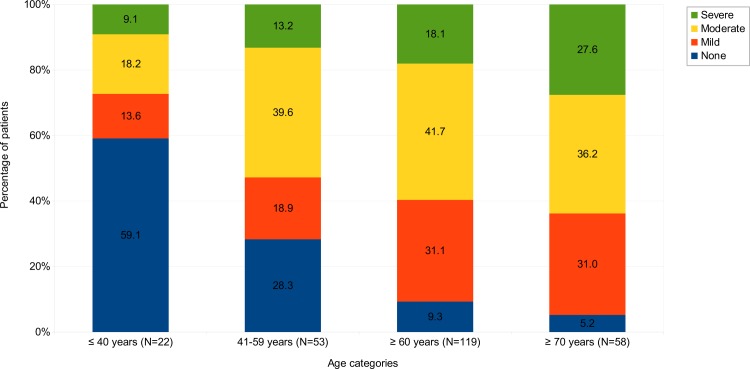
Distribution of ACE-27 categories in different age groups. Distribution of ACE-27 score of none, mild, moderate and severe comorbidities among four age groups in all 194 AML patients. 22 patients were younger than 41 years, 53 patients were between 41 and 59 years old, 119 patients were more than 60 and 58 patients more than 70 years old. Increasing age was significantly associated with higher ACE-27 score as analyzed by x^2^ test (*p* < .000).

Our results indicate an independent association of ACE-27-assessed comorbidity with OS while an independent influence of age was not observed. It has been shown consistently that patient age affects outcome in AML. This raised the question whether our analysis was biased by an abnormal distribution of comorbidities in the older patients. We therefore performed a subgroup analysis on distribution of ACE-27-assessed comorbidities in different age groups. Patients were separated into 4 groups: younger 40 years, 41 to 59 years of age, older than 60 and older than 70 years. As expected, increasing age was significantly associated with a higher comorbidity score (x² test; *p* < .000). Only 9.3% of patients *≥* 60 years and 5.2% *≥* 70 years had no comorbidities compared to 28.3% in the age group 41 to 59 years and 59.1% in the group < 40 years (Fig 4). A moderate or severe ACE score was found in 59.8% of patients ≥ 60 years and in 63.8% patients ≥ 70 years. The ACE-27 score was significantly associated in univariate analysis with OS in patients *≥* 60 years (*p* = .042), but not for patients < 60 years (*p* = .104). This suggests that comorbidities in addition to age impact OS in older AML patients.

## Discussion

We conducted a retrospective study to evaluate the impact of comorbidity on OS in patients with newly diagnosed AML. To measure severity of comorbidities we applied the HCT-CI, ACE-27 score and CIRS-G and analyzed the association of indices-based comorbidity scores with patient outcome. The ACE-27 score showed the most homogenous distribution in risk categorization. For all three indices an increase in comorbidity burden at initial diagnosis was associated with decreased survival. In multivariate models only the ACE-27 score was associated with OS in patients with AML. In patients receiving intensive curative treatment, increasing comorbidity, scored by the ACE-27, had an independent effect on survival besides cytogenetics and subsequent use of SCT. Intensively treated patients with a high ACE-27 score had a fourfold increased risk of death compared to patients without comorbidities.

Given the fact that the incidence of comorbidities increases with age comorbidity is an issue of growing importance due to the overall aging population [23]. Older patients have an average of 3 comorbidities in addition to their cancer [24]. Furthermore, the impact of comorbidity on survival, disease progression and treatment of patients with cancer has been widely studied, showing that increasing severity of comorbidities negatively affects survival [20]. Similar to other reports [9,10,25,26], older patients had a higher comorbidity burden in our study with the most frequent comorbidities comprising cardiovascular disease, infection, hepatic disorders and prior malignancy. Previous studies have shown cerebrovascular conditions, liver, renal and rheumatic disease to be main causes of non-leukemic death in AML [27]. Several comorbidity indices are available to measure comorbid conditions. However neither of these indices was initially designed for use in AML patients. The HCT-CI is one of the most commonly used index to study the impact of comorbidities on outcome in AML patients [9–11,13,14,25,26]. However, the HCT-CI was originally developed to predict outcome in patients undergoing hematopoietic SCT [13]. Since there exists no “gold standard” comorbidity index, we comparatively assessed the HCT-CI and two other established indices, the ACE-27 score and CIRS-G. The ACE-27 score was developed based on the frequency of occurrence of comorbidities in newly diagnosed cancer patients [20]. The CIRS is widely used to assess comorbidities among patients with CLL and defines them to be eligible or ineligible for intensive chemotherapy [28]. We could show, that all three indices were associated with OS in survival analysis. The HCT-CI seemed to separate risk groups better. But in adjusted models only the ACE-27 score was predictive for OS. Sorror and colleagues reported higher sensitivity of the HCT-CI compared to the ACE-27 score, resulting in higher predictive capacity for non-relapse mortality and survival of AML-patients undergoing SCT [14]. In contrast, our data did not show an association of HCT-CI with OS in multivariate analysis. Probably, the relatively small number of patients without comorbidities in the low-risk group in our study contributed to this disparate result.

The evaluation of comorbidities by the ACE-27 score has also been studied as a part of prognostic risk assessment in other hematological malignancies [15,16,29]. Similar to our findings, a high ACE-27 score was identified as an independent risk factor for worse OS in patients with MDS [15,16] and MPN [29]. For example, in a retrospective study of 600 patients with MDS the ACE-27 score accurately stratified the survival of patients according to the IPSS score [15].

For AML patients, a pretreatment higher comorbidity burden is associated with lower remission rate, increased early mortality and decreased survival [9–12,26]. For example, among 177 patients older than 60 years who received induction chemotherapy HCT-CI score of 0, 1–2 and ≥ 3 corresponded to early death (ED) rates (3%, 11% and 29%, respectively) and OS (45, 31 and 19 weeks, respectively) [10]. Similar data were reported in a retrospectively study of 233 adult AML patients treated with intensive chemotherapy [9]. In our study, scoring of pretreatment comorbidities using the ACE-27 score was predictive of OS. However, we could not confirm that a higher comorbidity score predicts ED in patients intensively treated with chemotherapy. We identified as single independent risk factor for ED an ECOG performance status ≥ 2. Our results are in line with other studies, reporting that ECOG is a stronger predictor for ED than comorbidity burden [30,31]. This suggest, that actual physical performance rather than comorbidities play a major role in treatment tolerance in the first days of intensive induction therapy.

In contrast to ED, long term survival as reflected by OS seems to depend on comorbidities rather than performance status, as we could saw no independent association of higher ECOG with OS in aggressively treated AML patients. Independent risk factors for OS were higher comorbidity burden assessed by ACE-27 score and poor risk cytogenetics. Similarly, in a single-center study of 120 patients older than 60 years of age treated with intensive chemotherapy HCT-CI score ≥ 3 was the single most significant predictor for worse OS, even outweighing karyotype [11].

Numerical age is a well established independent factor for survival in AML patients [5,6] and is commonly included in various prognostic models for outcome prediction in older adults with AML [8,12,17–19,32,33]. In our study, age did not exert a significant impact on OS in multivariate analysis when comorbidity scores were included. We conclude that the impact of numerical age on survival is probably partially exerted by an age-related comorbidity burden. Therefore, treatment decision should be based on age as well as comprehensive comorbidity assessment in regard to other patient- and disease-related factors.

There are several limitations to our study. The first is the retrospective nature of the data collection. Second, the long period of the study may bias the probability of survival particularly due to improvements in supportive treatment care. Third, the study was conducted as a single-institution analysis. Finally, our modest sample side may have limited power to investigate association in some points.

Despite this, our study clearly highlights the significant impact of comorbidities on the survival of patients with AML. Particularly for decision on intensive induction therapy, comorbidity assessment may be helpful. Our findings require prospective validation in multicenter randomized and non-interventional studies. Validated data on stratification based standardized comorbidity assessment will provide new tools for treatment decisions in patients with AML.

## Supporting Information

S1 Dataset The individual patient data. (PDF)

S1 Table Association of patient-related, treatment-related and disease-related factors with survival. (PDF)

S2 Table Multivariate models with factors predicting overall survival for the entire cohort and intensively treated patients with different ACE-groups. (PDF)
