# Supplementary material for: Value of Different Comorbidity Indices for Predicting Outcome in Patients with Acute Myeloid Leukemia
Source: PLoS One. 2016 Oct 12;11(10):e0164587. doi: 10.1371/journal.pone.0164587 (PMC5061362; doi:10.1371/journal.pone.0164587)
Supplement: S1 Dataset — (PDF) [file pone.0164587.s001.pdf]

S1 Dataset. The individual patient data.

ID	Age	Sex	LDH, U/L	WBC, /L	ECOG	Type	Cytogen	BMB, %	FAB	Tx	SCT	ED	CR	Status	OS, m	HCT-CI	CIRS-G	ACE-27
1	60	1	576	51.60	N/A	1	N/A	41	M2	1	1	1	1	2	32.85	2	2	0
2	63	1	1,272	3.00	N/A	2	3	20	M4	1	1	1	2	2	4.90	3	3	2
3	62	2	288	22.30	2	2	N/A	50	M4	1	1	1	1	2	5.88	3	2	2
4	56	1	282	8.60	N/A	2	N/A	N/A	N/A	1	1	1	1	2	36.24	3	3	3
5	39	2	468	15.70	N/A	1	N/A	30	M1	1	2	1	1	1	181.78	2	0	0
6	57	1	402	1.70	N/A	2	2	33	N/A	1	1	1	1	2	26.35	3	3	3
7	71	1	7,014	46.00	N/A	1	1	N/A	M3	1	1	1	1	2	14.65	3	2	2
8	58	2	840	16.00	1	1	2	85	M4	1	1	1	1	1	148.34	2	2	0
9	63	2	5,376	8.40	N/A	2	3	N/A	M1	1	1	1	1	2	19.61	3	2	2
10	69	2	463	0.80	N/A	2	N/A	40	M2	2	1	1	2	2	28.71	3	2	2
11	57	1	984	43.50	N/A	1	N/A	95	M4	1	1	1	1	2	22.90	2	2	0
12	57	1	696	9.10	1	1	1	70	M4	1	1	1	1	2	22.05	3	2	2
13	59	1	4,134	146.00	N/A	1	2	55	M2	2	1	1	2	2	2.86	2	2	0
14	74	2	480	41.60	0	1	N/A	N/A	M5	1	1	1	1	3	1.81	3	2	2
15	65	2	708	5.40	0	1	2	55	M1/2	1	1	1	1	2	18.07	2	2	1
16	59	1	1,236	13.50	N/A	1	2	70	M2/4	1	1	2	2	2	0.46	3	3	2
17	72	1	1,266	2.10	1	1	N/A	N/A	M1	1	1	1	1	2	7.29	3	2	3
18	59	1	1,446	2.60	1	1	3	30	M4eo	1	1	1	1	2	6.41	3	2	1
19	76	2	594	2.10	2	1	2	90	M0	1	1	1	1	2	32.23	2	2	1
20	54	2	1,460	209.90	2	1	N/A	N/A	M4	2	1	2	2	2	0.23	3	2	2
21	58	1	1,842	74.20	0	1	1	50	M4	1	1	1	1	1	78.06	2	3	3
22	40	2	576	26.80	N/A	1	1	N/A	M4eo	1	1	1	1	1	146.92	0	0	0
23	50	2	N/A	7.70	1	2	3	100	M4	1	1	2	2	2	3.06	3	3	2
24	68	2	624	7.98	N/A	1	N/A	85	M2	1	1	1	1	2	26.38	3	3	2
25	77	2	N/A	5.30	N/A	2	N/A	N/A	M5	2	1	2	2	2	0.36	3	3	3
26	32	2	276	2.30	N/A	2	N/A	N/A	M4	1	1	1	2	2	6.74	3	3	3
27	29	1	738	23.90	0	1	3	N/A	M4eo	1	1	1	1	3	4.30	2	0	0
28	70	1	330	1.70	0	2	2	5	M6	2	1	1	2	2	2.07	3	3	0
29	59	2	1,080	33.70	0	1	2	60	M4	1	1	1	1	1	130.60	2	2	0
30	77	2	642	2.10	2	2	3	90	M2	2	1	1	2	2	5.75	3	3	2
31	48	2	684	1.20	0	1	2	86	M6	1	1	1	1	3	41.13	2	2	0
32	35	1	1,374	20.60	0	1	2	N/A	M4	1	1	1	1	2	12.62	2	0	0

33	49	1	636	45.30	0	1	2	N/A	M4	1	1	2	2	2	0.46	2	2	0
34	49	1	822	9.90	0	2	N/A	5	M4	1	2	1	1	2	17.54	3	2	2
35	71	2	1,068	27.90	1	2	2	80	M4eo	1	1	1	2	2	11.89	3	2	2
36	19	1	1,848	28.00	0	1	2	80	M4	1	1	1	1	3	17.74	0	2	1
37	51	2	2,232	33.10	1	2	2	75	M4	1	1	1	1	3	6.67	3	3	2
38	63	1	N/A	0.80	1	2	3	30	N/A	1	1	1	2	2	3.25	3	2	2
39	43	1	1,890	7.40	0	1	2	40	M6	1	2	1	1	3	33.81	2	2	0
40	20	1	564	10.10	0	1	N/A	0	N/A	1	1	1	1	1	92.39	2	0	0
41	69	1	1,890	111.00	2	1	N/A	80	M2	2	1	1	2	2	0.43	3	3	1
42	33	2	660	17.50	0	1	2	N/A	M5	1	1	1	1	1	81.71	2	0	0
43	52	1	648	1.10	0	2	2	40	M4	1	2	1	1	1	113.71	3	3	2
44	70	1	N/A	163.00	1	1	2	90	M4	1	1	1	1	2	6.28	0	2	1
45	56	2	1,302	45.00	1	2	2	30	M4	1	2	1	1	3	49.87	3	2	2
46	53	2	1,692	8.80	1	1	2	80	M1	1	1	1	1	3	10.02	3	2	2
47	37	1	708	2.10	1	2	2	40	M2	1	2	1	1	2	21.09	3	2	2
48	66	1	1,026	4.00	2	2	2	40	M4	1	1	1	2	2	1.31	3	3	3
49	44	1	2,700	467.00	1	1	2	98	M1	1	2	1	1	2	96.53	2	0	0
50	62	2	4,758	48.70	N/A	1	N/A	80	M4	1	1	2	2	2	0.89	2	2	2
51	63	1	822	73.10	1	1	N/A	45	M2	1	1	1	1	2	15.61	2	2	0
52	61	2	564	2.40	1	2	2	30	M1	1	1	1	1	2	99.25	3	3	2
53	73	2	486	1.00	2	1	3	76	M0/1	2	1	1	2	2	0.16	2	2	2
54	66	1	1,608	96.20	1	1	2	45	M4	1	1	1	1	3	5.52	0	3	1
55	80	2	1,116	60.00	1	1	3	N/A	M2	2	1	1	2	2	0.00	2	2	2
56	73	1	1,716	1.80	2	2	N/A	N/A	M4	1	1	1	1	2	7.85	3	3	2
57	66	1	299	7.50	1	1	1	18	M2	1	1	1	1	2	19.81	3	3	2
58	77	2	60	11.40	1	1	2	60	M2	1	1	1	1	3	20.67	2	2	1
59	69	2	1,350	23.00	2	2	N/A	N/A	M4	2	1	1	2	2	0.13	3	3	2
60	68	1	606	11.50	1	1	2	90	M0	1	1	1	1	3	32.62	0	2	0
61	58	1	230	2.70	0	1	3	54	M6	1	2	1	1	2	15.24	0	2	1
62	34	2	317	11.40	0	1	2	80	M2	1	2	1	1	1	96.76	2	2	0
63	69	1	429	0.50	1	2	N/A	30	N/A	2	1	1	2	3	0.13	3	2	3
64	77	2	341	31.10	N/A	2	2	20	M5	2	1	1	2	3	23.23	3	3	3
65	48	1	606	4.60	1	1	3	40	M2	1	2	1	1	2	12.29	2	2	2
66	18	1	1,230	16.50	1	1	2	80	M2	1	1	1	1	3	5.62	2	0	0
67	45	1	420	9.30	1	1	2	60	M4	1	2	1	1	1	90.12	2	2	0

68	62	2	288	1.00	0	2	2	50	M2	1	2	1	1	3	9.20	3	3	2
69	54	2	1,477	88.41	1	2	3	20	M4	1	1	1	2	2	8.94	3	3	3
70	61	2	329	7.60	0	1	3	0	M4	1	2	1	1	2	4.60	3	3	3
71	90	1	563	45.90	3	1	2	40	M2	2	1	1	2	2	0.36	2	3	3
72	64	1	372	15.00	N/A	2	2	60	M2	1	2	1	1	1	91.43	3	2	2
73	47	2	449	5.25	1	2	1	40	M2	1	2	1	1	1	69.75	3	3	2
74	68	2	654	3.50	1	2	2	80	M4	1	2	1	1	1	69.39	3	3	2
75	66	2	754	120.00	2	1	N/A	30	M0	1	1	1	2	2	4.47	3	3	1
76	60	2	600	3.20	1	1	1	60	M3	1	1	1	N/A	3	0.76	3	2	2
77	75	2	2,145	13.08	3	1	2	90	M4	1	1	2	2	2	0.10	3	3	3
78	72	2	1,331	113.50	1	1	3	80	M2	1	1	1	1	2	17.61	2	2	1
79	76	2	N/A	1.61	2	1	3	N/A	M4	1	1	1	1	3	2.00	2	3	2
80	40	2	970	116.80	0	1	2	90	M4	1	2	1	1	2	6.28	3	2	1
81	67	2	N/A	1.78	0	2	3	40	M2	1	2	1	1	1	71.79	3	2	1
82	76	2	211	2.03	1	2	N/A	64	M1	1	1	1	1	3	61.34	0	2	1
83	63	2	595	N/A	0	1	2	N/A	M2	1	1	1	1	3	54.05	0	0	0
84	69	2	1,200	183.00	2	1	2	87	M2	1	1	1	1	2	37.72	3	3	1
85	68	1	398	134.00	0	1	2	90	M0	1	1	1	2	2	9.20	3	3	1
86	53	1	678	300.00	1	1	2	87	M1	1	1	1	1	3	28.02	3	2	1
87	65	2	N/A	150.00	1	1	2	0	M2	1	2	1	1	1	67.81	0	2	1
88	75	2	482	3.97	1	1	2	45	M2	1	1	1	2	3	10.58	3	3	3
89	69	1	868	55.54	1	2	2	50	M5	1	1	1	1	2	12.58	3	3	2
90	65	1	627	125.98	2	1	2	83	M5	1	1	2	2	2	0.10	2	2	1
91	60	1	224	12.57	0	2	3	30	M1	1	1	2	2	2	2.10	3	3	2
92	73	1	725	184.00	1	1	2	90	M2	1	1	1	2	3	4.63	2	2	1
93	79	1	217	4.49	1	1	N/A	80	N/A	1	1	1	2	3	32.39	3	3	3
94	48	2	644	114.10	1	1	2	50	M4	1	1	1	1	2	2.14	2	2	2
95	66	2	286	0.99	1	1	1	30	M2	1	1	1	1	2	29.17	2	2	1
96	79	2	369	49.67	0	1	N/A	100	M2	1	1	2	2	2	0.92	2	2	2
97	72	1	334	24.06	2	2	N/A	15	M4	1	1	1	1	1	39.52	3	3	3
98	58	2	310	17.10	1	1	2	14	M2	1	1	1	2	2	8.90	3	3	3
99	67	1	256	2.00	1	1	3	40	M2	1	2	1	1	2	16.62	2	3	1
100	56	1	206	8.53	1	1	1	60	M2	1	2	1	1	1	62.42	2	2	1
101	74	1	414	124.12	2	2	N/A	60	M4	1	1	1	2	2	3.61	3	3	3
102	75	1	581	13.25	0	1	N/A	40	M2	1	1	1	1	1	22.67	2	2	1

103	74	1	955	55.36	1	2	N/A	50	M2	1	1	1	2	2	0.85	3	3	3
104	67	1	10,312	24.93	3	1	N/A	50	M5	1	1	1	2	2	2.07	3	3	1
105	67	2	801	19.07	1	1	2	90	M4	1	1	1	1	2	8.08	2	2	1
106	67	1	433	24.82	1	1	2	90	M5	1	1	1	1	2	7.56	3	3	3
107	75	1	248	386.00	1	1	2	80	M2	1	1	1	1	3	9.03	2	2	1
108	82	2	1,478	1.30	2	2	2	60	M0	2	1	1	1	2	2.63	3	3	3
109	56	1	427	51.89	1	1	2	75	M2	1	2	1	1	2	10.45	2	2	0
110	64	1	377	7.70	1	2	3	30	N/A	1	2	1	1	2	27.43	3	3	2
111	62	2	20	105.00	0	2	2	90	M2	1	1	1	2	2	25.56	2	2	2
112	65	1	568	175.51	1	1	2	75	M1	1	1	1	1	1	51.58	3	2	0
113	53	2	323	8.41	1	1	1	20	M4	1	2	1	1	1	26.18	3	3	2
114	44	2	335	6.00	0	2	3	100	M7	1	2	1	1	2	4.17	3	2	1
115	69	1	4,864	18.60	0	2	3	N/A	N/A	1	1	2	2	2	0.16	3	2	2
116	78	1	514	50.81	1	1	N/A	90	M0	1	1	1	1	2	19.78	2	2	1
117	74	2	500	35.61	1	2	N/A	0	M4	1	1	1	1	2	16.23	3	3	3
118	52	1	238	2.94	0	2	3	70	M2	1	2	1	1	2	50.66	3	2	2
119	69	1	2,486	202.19	0	1	N/A	90	M4	1	1	1	2	2	0.03	2	2	2
120	79	1	997	56.16	2	2	N/A	N/A	N/A	1	1	2	2	2	1.91	3	3	3
121	81	2	1,340	211.00	2	2	2	100	M2	1	1	1	N/A	1	6.64	3	2	2
122	42	2	641	32.68	1	2	2	76	M1	1	1	1	2	2	0.76	3	3	3
123	73	2	574	28.80	2	2	2	60	M2	1	1	1	1	2	47.97	3	3	1
124	78	1	239	1.88	1	2	2	45	N/A	1	1	1	1	2	40.08	3	3	3
125	70	2	868	8.82	1	2	3	40	M2	1	1	1	1	2	16.92	3	3	2
126	47	2	498	1.11	1	1	2	30	M2	1	1	1	1	2	15.80	3	2	0
127	72	1	319	16.44	1	2	2	40	M1/2	1	1	1	2	1	10.61	3	3	2
128	72	2	1,336	97.00	0	1	N/A	90	M5	1	1	2	2	2	0.43	3	3	2
129	53	1	283	10.23	1	2	2	5	M2	1	1	1	1	1	42.41	3	3	3
130	76	2	2,146	28.17	3	1	N/A	95	M0	1	1	2	2	2	0.43	3	3	2
131	81	2	1,231	42.90	1	2	N/A	66	M0	1	1	2	2	2	0.62	3	3	2
132	63	1	555	74.00	1	1	2	70	M2	1	1	1	1	2	22.11	0	3	1
133	63	2	706	43.66	1	1	3	80	M2	1	1	1	2	2	10.94	0	2	0
134	67	1	337	24.30	1	1	2	89	M1	1	2	1	1	1	38.57	2	3	0
135	51	1	N/A	15.30	1	1	3	90	M5	1	1	1	1	2	9.10	2	2	1
136	79	2	232	23.45	1	1	N/A	80	M2	2	1	1	2	2	0.99	0	2	0
137	61	1	1,247	38.87	0	2	N/A	80	M0	1	1	1	2	2	1.77	3	3	2

138	70	1	435	43.63	1	1	N/A	76	M4	1	1	1	1	1	36.47	3	3	1
139	49	2	199	1.06	0	1	1	24	M2	1	1	1	1	1	21.26	3	2	2
140	58	1	1,165	19.23	1	1	3	55	M1	1	1	1	1	2	25.03	3	2	1
141	45	1	340	13.23	0	2	2	80	M4	1	2	1	1	2	6.24	3	2	2
142	56	2	2,005	62.57	0	1	1	60	M4	1	1	1	1	1	26.74	2	2	1
143	46	2	N/A	2.70	0	1	3	24	M2	1	2	1	1	1	26.45	2	2	0
144	78	2	262	1.34	1	2	N/A	30	M4	1	1	1	1	1	10.09	2	2	1
145	51	2	1,567	49.18	N/A	1	2	30	M4	1	1	1	1	2	18.46	2	2	0
146	66	1	181	3.12	2	2	2	40	M0/1	1	1	1	1	2	9.00	3	3	2
147	68	2	567	13.30	N/A	1	2	20	M2	1	1	1	1	2	1.74	3	2	2
148	66	1	319	35.78	0	1	2	90	M5	1	2	1	1	2	18.79	3	2	1
149	74	2	2,784	21.20	1	1	1	100	M1/2	1	1	1	1	1	3.29	3	2	1
150	64	1	571	162.31	2	1	2	50	M1/2	1	2	1	1	2	13.67	2	3	1
151	69	1	1,229	8.18	2	1	N/A	N/A	N/A	1	1	2	2	2	12.62	2	3	1
152	31	1	229	1.60	0	2	2	66	M0	1	2	1	1	1	14.13	3	2	2
153	43	1	N/A	22.09	1	2	3	20	M4	1	1	1	N/A	1	0.99	3	2	2
154	71	2	636	5.86	1	1	1	27	M1/2	1	1	1	1	2	3.48	3	3	2
155	60	1	3,053	36.60	1	1	2	100	M4	1	1	1	1	2	2.73	3	2	0
156	79	1	370	527.00	N/A	1	N/A	30	M4	2	1	2	2	2	0.95	3	0	0
157	28	1	277	3.29	N/A	1	2	35	M2	1	1	1	1	1	23.98	2	2	2
158	64	2	2,005	7.50	1	1	2	22	M0/1	1	1	1	1	2	8.84	3	3	2
159	79	2	558	93.09	1	1	N/A	85	M1	1	1	1	1	1	3.12	3	3	1
160	66	1	197	1.39	0	2	3	52	M2	1	2	1	1	1	26.25	3	2	2
161	53	1	221	8.02	1	2	3	30	M2	1	1	1	1	2	3.48	3	2	2
162	51	2	203	3.30	0	2	3	70	M1	1	2	1	1	2	3.38	3	2	2
163	31	1	610	33.86	0	1	3	80	M1	1	2	1	2	1	17.45	2	2	0
164	22	2	279	14.92	0	1	1	80	M3	1	1	1	1	1	4.01	2	0	0
165	52	2	263	3.06	0	1	1	N/A	M6	1	2	1	1	2	10.58	3	3	1
166	52	1	368	130.00	1	2	2	56	M4	1	2	1	2	2	16.49	3	2	2
167	23	1	215	0.76	0	1	3	35	M6	1	2	1	1	1	17.51	0	0	0
168	39	2	372	20.00	0	1	1	N/A	M3	1	1	1	1	1	15.47	3	2	2
169	75	2	617	9.20	N/A	1	2	N/A	N/A	1	1	1	1	2	7.72	3	2	1
170	68	2	334	22.98	N/A	1	N/A	70	M5	1	1	1	1	2	6.90	2	2	2
171	53	1	297	4.56	N/A	1	2	N/A	N/A	1	1	2	2	2	0.46	2	2	0
172	55	2	389	48.56	N/A	1	2	83	N/A	1	1	1	1	1	10.78	2	2	0

173	63	1	1,485	88.05	N/A	2	N/A	100	N/A	1	1	2	2	2	0.95	3	3	3
174	62	1	397	145.31	N/A	1	3	80	M4	1	2	1	1	1	16.03	3	3	1
175	69	1	550	84.56	N/A	1	3	85	N/A	2	1	1	N/A	1	0.36	2	3	1
176	78	1	242	1.96	0	2	3	50	M2	1	1	1	1	1	4.76	3	3	2
177	24	2	148	1.86	0	2	3	34	M2	1	2	1	1	1	10.38	3	3	3
178	77	2	400	23.57	1	1	N/A	85	M2	1	1	1	1	1	8.51	3	3	1
179	31	1	308	1.01	0	1	2	90	M3	1	1	1	1	1	3.75	2	2	0
180	65	2	472	200.00	0	1	2	96	M2	1	2	1	1	1	7.89	3	3	2
181	72	2	206	5.32	0	2	2	75	M2	1	2	1	2	1	9.07	3	3	2
182	38	1	428	37.70	0	1	2	60	M4	1	1	1	1	1	5.26	2	2	0
183	58	1	545	44.85	0	1	2	80	M1/4	1	2	1	2	1	9.59	2	2	1
184	77	1	311	25.00	1	1	N/A	70	M4	1	1	1	1	1	3.35	2	3	1
185	78	2	354	18.24	2	2	N/A	70	N/A	1	1	1	1	1	7.52	3	3	2
186	77	1	252	1.80	1	2	2	19	N/A	2	1	1	1	1	2.04	3	3	2
187	73	1	287	14.53	N/A	1	3	60	M4	2	1	1	2	2	1.31	3	3	1
188	74	2	479	2.00	N/A	2	2	30	N/A	1	1	1	2	1	5.45	3	3	2
189	69	1	245	19.79	0	1	1	90	M4	1	1	1	1	1	2.69	3	3	2
190	63	2	1,148	37.15	1	1	2	80	M4/5	1	1	1	1	1	3.52	3	3	3
191	59	1	585	152.65	N/A	1	N/A	85	M4	2	1	2	2	2	0.23	3	3	2
192	72	1	608	55.81	N/A	2	N/A	42	M4	1	1	1	2	1	1.41	3	3	3
193	35	1	1,113	335.00	N/A	1	1	86	M4	1	1	1	2	2	0.00	3	2	1
194	88	1	275	1.99	2	2	2	39	M4	2	1	1	2	1	0.33	3	3	3

LDH, lactate dehydrogenase; WBC, white blood cells; ECOG, Eastern Cooperative Oncology Group; Cytogen, Cytogenetics; BMB, bone marrow blasts; FAB, French-American-British; Tx, therapy regimen; SCT, stem cell transplantation; ED, early death; CR, complete remission; OS, m, overall survival in months; HCT-CI, Hematopoetic cell transplantation comorbidity index; CIRS-G, Cumulative Illness Rating Scale for Geriatrics; ACE-27, Adult Comorbidity Evaluation-27; N/A, not available; Sex, 1= male; 2= female; Type, 1= de novo, 2= secondary; Cytogenetics according to ELN guidelines [1], 1= good; 2= intermediate; 3= poor; Tx, 1= intensive, 2= palliative; SCT, 1= no SCT, 2= SCT; ED, 1= no ED; 2= ED; CR, 1= CR, 2= no CR; Status, 1= alive, 2= death, 3= lost to follow up; HCT-CI, 1= 0; 2= 1-2; 3= 3 and more; CRIS-G, 1= 0, 2= 1-6; 3= >6.
