# Supplementary material for: Value of Different Comorbidity Indices for Predicting Outcome in Patients with Acute Myeloid Leukemia
Source: PLoS One. 2016 Oct 12;11(10):e0164587. doi: 10.1371/journal.pone.0164587 (PMC5061362; doi:10.1371/journal.pone.0164587)
Supplement: S1 Table — (PDF) [file pone.0164587.s002.pdf]

Table S1. Association of patient-related, treatment-related and disease-related factors with survival.

Variable		Total no. of patients	Death N (%)	Median survival (months)	*p**
Age, years	< 60	75	37 (49.3)	25	.003
	≥ 60	119	119 (63.0)	13	
Sex	Male	102	62 (60.8)	16	.339
	Female	92	50 (54.8)	18	
ECOG	0-1	131	64 (49.9)	21	.000
	≥ 2	29	24 (82.8)	4	
Regimen	Intensive CTx	173	96 (55.5)	19	.000
	Palliative CTx	21	16 (76.2)	1	
SCT	No	151	94 (62.3)	12	.000
	Yes	43	18 (41.9)	96	
Cytogenetics	Good	19	7 (36.8)	29	.009
	Intermediate	87	44 (50.6)	25	
	Poor	38	26 (68.4)	12	
AML type	De novo	123	68 (53.3)	18	.457
	Secondary	71	44 (62.0)	16	
WBC, /L	≤ 30×10^9	123	66 (53.7)	21	.021
	> 30×10^9	71	46 (64.8)	14	
LDH, U/l	≤ 700	119	58 (48.7)	27	.000
	> 700	65	49 (75.4)	8	
BM blast, %	≤ 30	37	22 (59.5)	17	.994
	> 30	132	72 (54.5)	21	

ECOG, Eastern Cooperative Oncology Group; CTx, chemotherapy; SCT, stem cell transplantation; WBC, white blood cell; LDH, lactate dehydrogenase; BM, bone marrow.

* *p*-values from univariate long-rank test.
