# Supplementary material for: Value of Different Comorbidity Indices for Predicting Outcome in Patients with Acute Myeloid Leukemia
Source: PLoS One. 2016 Oct 12;11(10):e0164587. doi: 10.1371/journal.pone.0164587 (PMC5061362; doi:10.1371/journal.pone.0164587)
Supplement: S2 Table — (PDF) [file pone.0164587.s003.pdf]

Table S2. Multivariate models with factors predicting overall survival for the entire cohort and intensively treated patients with different ACE-groups.

	entire cohort (N=194)			Intensively treated patients (N=137)		
Variable	HR*	95% CI	*p*	HR*	95% CI	*p*
Age, years						
< 60	Ref			Ref		
≥ 60	1.43	0.79-2.59	NS	1.14	0.62-2.11	NS
ACE-27 score						
0 (no comorbidity)	Ref			Ref		
1-3 (any comorbidity)	2.49	1.13-5.49	.024	3.34	1.47-7.59	.004
Cytogenetics						
Good	Ref			Ref		
Intermediate	1.75	0.65-4.66	NS	2.10	0.81-5.47	NS
Poor	4.25	1.58-11.45	.004	4.91	1.77-13.62	.002
ECOG						
0-1	Ref			Ref		
≥ 2	2.73	1.36-5.50	.005	1.75	0.75-4.07	NS
SCT						
Yes	---			Ref		
No	---			1.94	1.06-3.54	.031
LDH, U/l						
≤ 700	Ref			Ref		
> 700	1.28	0.71-2.32	NS	1.05	0.53-2.08	NS
WBC, /L						
≤ $30x10^9$	Ref			Ref		
> $30x10^9$	1.45	0.84-2.53	NS	1.36	0.77-2.42	NS

HR, hazard ratio; CI, confidence interval; ACE-27, Adult Comorbidity Evaluation-27; ECOG, Eastern Cooperative Oncology Group; WBC, white blood cell; LDH, lactate dehydrogenase; SCT, stem cell transplantation; NS, not significant; Ref, reference.

--- indicates not applicable

*HR was adjusted to age, ACE-27, ECOG, cytogenetics, SCT, WBC and LDH.
